# Two-Layer Microstructures Fabricated by One-Step Anisotropic Wet Etching of Si in KOH Solution †

**DOI:** 10.3390/mi7020019

**Published:** 2016-01-25

**Authors:** Han Lu, Hua Zhang, Mingliang Jin, Tao He, Guofu Zhou, Lingling Shui

**Affiliations:** Institute of Electronic Paper Displays, South China Academy of Advanced Optoelectronics, South China Normal University, Guangzhou 510006, China; hanlunkq@gmail.com (H.L.); huazhang717@gmail.com (H.Z.); taohewhh@gmail.com (T.H.); zhougf@scnu.edu.cn (G.Z.)

**Keywords:** wet etching, potassium hydroxide, Si, pattern

## Abstract

Anisotropic etching of silicon in potassium hydroxide (KOH) is an important technology in micromachining. The residue deposition from KOH etching of Si is typically regarded as a disadvantage of this technology. In this report, we make use of this residue as a second masking layer to fabricate two-layer complex structures. Square patterns with size in the range of 15–150 μm and gap distance of 5 μm have been designed and tested. The residue masking layer appears when the substrate is over-etched in hydrofluoric acid (HF) solution over a threshold. The two-layer structures of micropyramids surrounded by wall-like structures are obtained according to the two different masking layers of SiO_2_ and residue. The residue masking layer is stable and can survive over KOH etching for long time to achieve deep Si etching. The process parameters of etchant concentration, temperature, etching time and pattern size have been investigated. With well-controlled two-layer structures, useful structures could be designed for applications in plasmonic and microfluidic devices in the future.

## 1. Introduction

Anisotropic etching of silicon in alkali metal hydroxides aqueous solutions (e.g., KOH) is an important technology in micromachining [1]. Micro- and nano-structures on substrate have been widely applied in solar cells, superhydrophobic surface and plasmonics [2,3,4,5,6]. Different applications require the features at a different shape and size range.

The formation of micro- and nano-pyramids on the Si surfaces are well known fabricated by this anisotropic etching process [5,6]. KOH has often been selected as the etchant according to its advantages of easy-preparation, non-toxicity, cost-effective and fast-etching [7]. For anisotropic etching of Si, the KOH concentration and etching temperature are key parameters, especially for the structures at nanometer scale. For most micron scale fabrication, the KOH concentration varies from 25–50 wt % and temperature range of 50–85 °C [1,5,6,7,8,9,10,11,12,13,14,15,16]. The higher etchant concentration and reaction temperature guarantee that the etching products are dissolved fast without hiding the continuous etching. However, if people would like to precisely control the etching process, such as nanostructure by KOH etching, the lower KOH concentration and lower temperature are required [6].

The residue deposition from KOH etching of Si is well known [8], which is typically considered as a disadvantage of this fabrication technology. In this work, we make use of the residue as a second masking layer for fabrication of two-layer microstructures. Square arrays with different pattern size from 15 to 150 μm and gap distance of 5 μm have been designed and tested. Normal micropyramids can be fabricated by well-controlled SiO_2_ and Si etched in HF and KOH solutions. When the substrate is over-etched in HF to achieve larger gaps, the second layer wall-like structures appear among the first layer micropyramids. Carefully control the fabrication parameters, the two-layer structure dimensions can be tuned precisely. The residue masking layer is strong and stable, can survive longer than the SiO_2_ masking layer for KOH etching, which in the end induces inversed structures. 

Two-layer micro/nanostructures are important to achieve step-emulsification microfluidic devices [17,18] and superhydrophobic surfaces [19,20]. With standard microfabrication technology, it typically requires twice lithography and wet-etching to achieve two-layer microstructures. By this method, two-layer microstructures can be fabricated in simple one-step lithography and wet-etching process, which is very useful for fabrication of functional devices for plasmonics and microfluidics applications.

## 2. Experimental Section

P-type <100> 4” silicon wafer with 100 nm SiO_2_ (Lijing Optoelctronics Co. Ltd, Suzhou, China) was used as the substrate for surface patterning and etching. The Si wafers were ultrasonically cleaned in Deionized (DI) water for 15 min, immersed in Piranha solution for 15 min and then thoroughly rinsed by DI water. Photolithography was done by spin coating (Smart Coater 100, Best Tools, LLC, St Louis, MO, USA) photoresist SUN-120P (Suntific Microelectronic Materials Co. Ltd, Weifang, China) at 3000 rpm for 60 s, exposing using an aligner (URE-200/35, Institute of Optics and Elctronics, Chendu, China) for 30 s, and developing in 0.4 wt % KOH at 25 °C for 2 min. The wafer with photoresist was then rinsed using DI water and dried using nitrogen gun, and then hard baked on a hot plate (EH20B, Lab Tech, Beijing, China) at 120 °C for 15 min. SiO_2_ etching was performed in 10 wt % HF (Guangzhou Chemistry, Guangzhou, China) solution, and anisotropic Si etching was completed in KOH (Zhiyuan Chemistry, Tianjin, China) solution. All chemicals were used as received without further treatment. Desktop scanning electron microscope (SEM) (Phenom G2 Pro, Phenom-World, Eindhoven, The Netherlands) and Field Emission-SEM (FE-SEM) (ZEISS-Ultra55, Carl Zeiss AG, Oberkochen, Germany) were used to visualize micro- and nano-structures and take images. Contact angle was measured using OCS 15pro (Dataphysics, Stuttgart, Germany).

## 3. Results and Discussion

Pyramids in the range of micrometer to nanometer size can be fabricated by anisotropic etching of silicon surface in KOH etchant solution. The formation of pyramids is neither related to any specific KOH supplier nor to mask or lithography problems [1]. Usually, the KOH etched pyramids are of exact geometric shape. Two types of pyramids can be observed: rectangular base or octagonal base, depending on the experimental conditions [21]. 

In general, pyramids are obtained according to the anisotropic etching of <100> and <110> plane in KOH solution. In this work, we have designed the square patterns in the range of 15 to 150 μm with gap distance of 5 μm. By controlling the etching time in HF solution, the opening of SiO_2_ (first masking layer for KOH etching) varies with etching time. We have obtained simple micropyramids structure by strictly control the etching time in HF solution. However, prolonged etching time in HF solution expand the opening size of the SiO_2_ on Si substrate, which causes fast reaction with large amount of products which will deposit on Si surface serving as a second masking layer to produce a second layer of wall-like structures among the first layer micropyramids.

### 3.1. Two Types of Microstructures Obtained in One-Step Wet Etching

**Figure 1:**
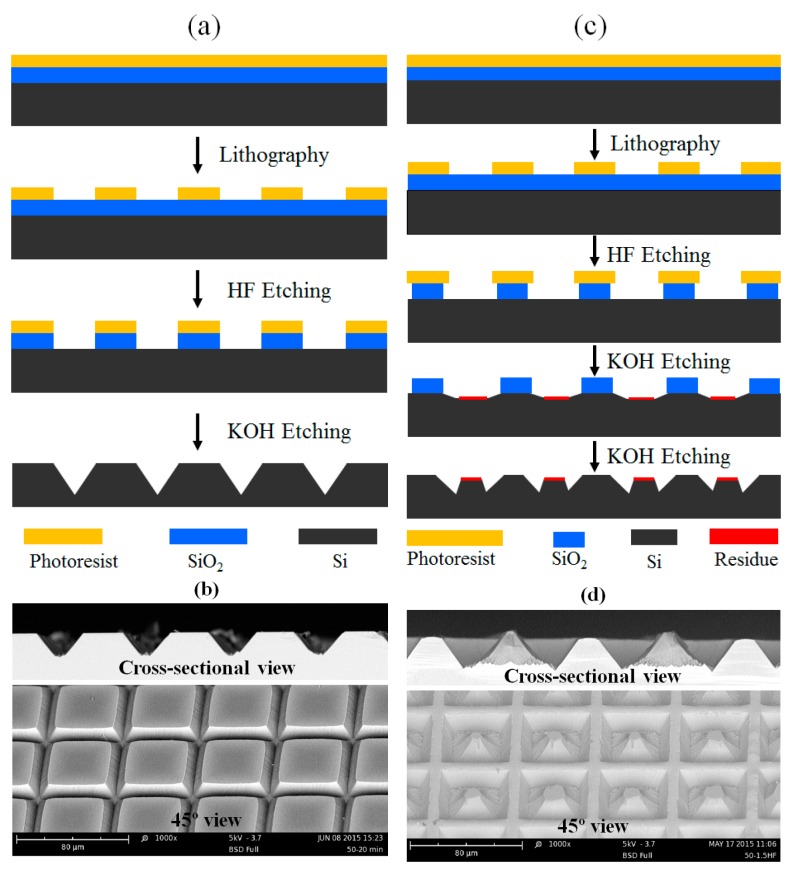
Schematic drawing of the fabrication process without (**a**) or with (**c**) the residue as second masking layer. (**b**) SEM images of cross-sectional view (top) and 45° view (bottom) of the fabricated one-layer micropyramids. (**d**) SEM images of cross-sectional view (top) and 45° view (bottom) of the fabricated two-layer micropyramids surrounded by walls.

In our experiments, we found that with the variation of pattern size and etching conditions different types of patterns have been obtained. Figure 1a,c represents the schematic cross-sectional view of the fabrication process of one-layer micropyramids and two-layer with first layer micropyramids surrounded by second layer wall-like structures, respectively. Figure 1b,d shows the SEM images of the fabricated structures.

**Figure 2:**
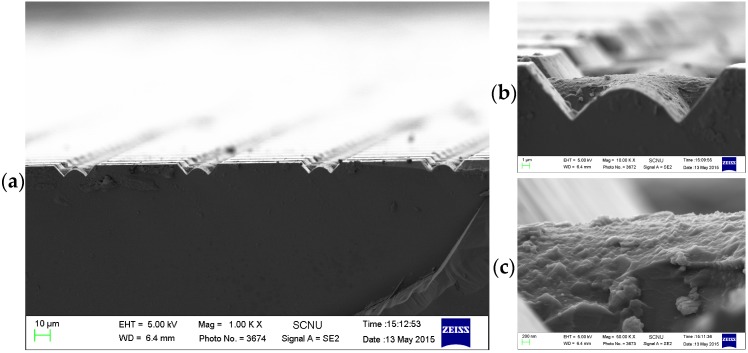
SEM images of a two-layer structures (**a**,**b**), and the residue substance on the wall-like structure surface (**c**). The pattern size was 75 μm × 75 μm with 5 μm gap distance. The substrate was etched in 10 wt % HF for 1.5 min, and then in 10 wt % KOH solution for 30 min at 70 °C.

The simple micropyramids are obtained by anisotropic etching silicon via the 5 μm opening in KOH solution, micropyramids connected with each other via the valleys with the same shape and size, as shown in Figure 1b. By carefully analyzing SEM image in Figure 1d (top), we can clearly see that the height and width of the neighboring micropyramids are different; however, all even micropyramids show the same shape and size and all odd micropyramids show the same shape and size. The bottom image in Figure 1d clearly show that two types of microstructures were obtained in our experiments. The first layer of micropyramids were obtained by anisotropic etching silicon substrate via the opened Si by HF etching of SiO_2_, and the second layer of wall-like structures surrounding the micropyramids were obtained according to the second masking layer from the reaction products deposition. The second residue masking layer is mainly Si(OH)*x* from the reaction of KOH and Si, which could be easily removed if the sample was dipped in HF solution again. Figure 2 shows the high resolution SEM image of the substance deposited on the wall-like structure surface.

We have designed and tested the square patterns with side length of 15, 30, 50, 65, 75, 100 and 150 μm, and all gap distance of 5 μm. Both simple one-layer micropyramids and complex two-layer structures of micropyramids surrounded by the wall structures have been observed for all these designed structures. In general, the one-layer micropyramids appear when the etching time in HF solution (*t*_HF_) is ≤ 1.0 min. As soon as *t*_HF_ ≥ 1.5 min, the two-layer structures started to appear.

### 3.2. HF Etching Time Effect on the Two-Layer Structures 

**Figure 3:**
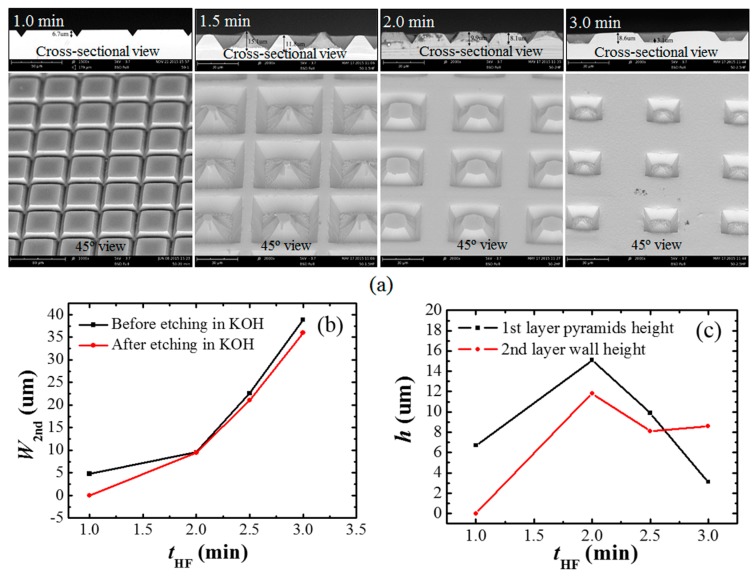
(**a**) SEM images of the fabricated structures at different *t*_HF_ (top: cross-sectional view, bottom: 45° view). (**b**) The second layer wall width (*W*_2nd_) varies with *t*_HF_. (**c**) Height (*h*) of the first layer micropyramids and the second layer walls changes with *t*_HF_. The sample patterns are 50 μm × 50 μm square with gap distance of 5 μm. The samples were etched in HF solution for 1.0, 1.5, 2.0 and 2.5 min. All sample substrates were etched in 10 wt % KOH solution for 30 min at 70 °C.

As discussed in the previous section, the two-layer structures appear according to the over etching in HF solution. We have tested the HF etching time (*t*_HF_) effect on the first layer micropyramids and second layer wall-like structures using the sample of 50 μm × 50 μm square patterns with 5 μm gap distance, as shown in Figure 3. Figure 3a is the SEM images of the fabricated structure when the samples were immersed in HF for different time. Figure 3b,c shows the variation of the two-layer structure width and height with *t*_HF_.

The simple one-layer micropyramids were obtained when the sample was etched in HF for 1.0 min. As *t*_HF_ increased to 1.5, 2.0 and 3.0 min, obvious two-layer structures were observed. The height of the micropyramids (first layer) and wall-like structures (second layer) is 15.1 and 11.8 μm when *t*_HF_ = 1.5 min, 9.9 and 8.1 μm when *t*_HF_ = 2.0 min, 3.1 and 8.6 μm when *t*_HF_ = 3.0 min. Therefore, the height difference between first layer and second layer is 3.3, 1.8 and 5.5 μm for *t*_HF_ of 1.5, 2.0 and 3.0 min, respectively. The maximum second layer wall-like structures were obtained at *t*_HF_ = 1.5 min. A special structure was obtained at *t*_HF_ = 3.0 min, in which the micropyramids were smaller than the wall structures. As seen from the details of the SEM images, the SiO_2_ layer disappeared during the KOH etching, leaving its covered Si completely open for KOH etching which caused the micropyramids to shrink. However, the wall-like structures were stable over all etching processes, showing strong and wide wall structures. Therefore, we can conclude that the two-layer complex structures are produced according to the two masking layers: the first SiO_2_ masking layer and the second residue masking layer from the quick accumulation of reaction products of KOH and Si in the open area, as demonstrated in Figure 1c.

### 3.3. KOH Concentration and Etching Time Effect on Two-Layer Structures 

**Figure 4:**
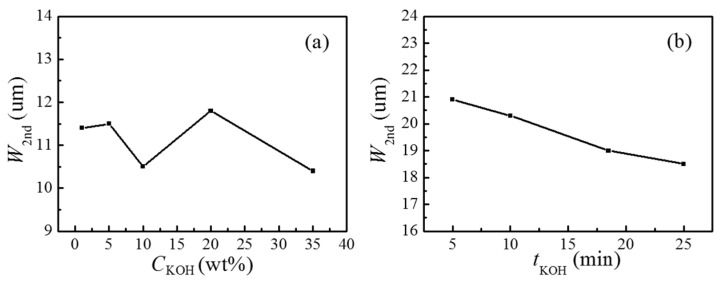
(**a**) Width of the second layer walls (*W*_2nd_) varies with KOH concentration(*C*_KOH_). (**b**) Width of the second layer walls (*W*_2nd_) changes with etching time in KOH solution (*t*_KOH_). The sample patterns are 50 μm × 50 μm square with gap distance of 5 μm. Each data point was obtained by averaging the values from three samples. All samples were etched in HF solution for 1.5 min. The etching time and temperature was 30 min and 70 °C for (**a**). The KOH concentration was 10 wt % KOH and etching temperature was 70 °C for (**b**).

The effect of KOH concentration (*C*_KOH_) and etching time in KOH solution (*t*_KOH_) on the second layer wall structures have also been investigated, as shown in Figure 4. As the KOH concentration increases, the wall width does not change significantly, as shown in Figure 4a. The wall width slightly decreases with the etching time in KOH, as shown in Figure 4b.

### 3.4. Shapes of Micropyramids

**Figure 5:**
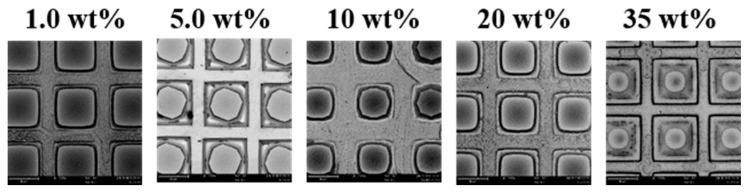
First layer micropyramids shape varies with KOH concentration. All samples were 50 μm × 50 μm square patterns with gap of 5 μm. The samples were first etched in 10 wt % HF solution for 2.0 min. The KOH etching time was 10 min and temperature was 70 °C.

Usually the KOH etched pyramids are of exact geometric shape. Two types of pyramids can normally be observed: rectangular base or octagonal base, depending on the experimental conditions [21]. In our experiments, we have also observed the rectangular and octagonal shapes for the first layer micropyramids directly etched via the opening of the SiO_2_ layer. The samples with the same pattern size (50 μm × 50 μm square with 5 μm gap distance) was selected to investigate the shape of the micropyramids. The samples were all etched in 10 wt % HF solution for 2.0 min, and then moved to KOH solutions to etch for 10 min at 70 °C. As shown in Figure 5, two-layer structures are obtained for all samples; however the shape of the micropyramid tips changes with process conditions. The shape is square, octagonal, mixed square and octagonal, square with round-corners and round at the KOH concentration of 1.0, 5.0, 10, 20 and 35 wt %, respectively. This is according to the plane selectivity and etching speed, which is similar to simple micropyramid structures without the second layer structures [9]. 

## 4. Conclusions

In this work, we have designed micropatterns for fabrication of micropyramids on silicon substrate with SiO_2_ as masking layer. Simple one-layer micropyramids have been obtained with controlled etching in HF solution to open the etching access holes at around 5 μm. Two-layer complex structures of first layer micropyramids surrounded by second layer wall-like structures were obtained by over-etching the substrate in HF solution. With the square pattern size in the range of 15 to 150 μm with 5 μm gap distance, reproducible two-layer structures have been obtained in a wide range of fabrication conditions. As an example, 50 μm × 50 μm patterns with 5 μm gap, the two layer micropyramids appeared when HF etching time was more than 1.5 min. With increasing the etching time in HF, the second layer wall-like structure width and height increases, however the first layer micropyramids height and width decreases. KOH concentrate does not affect the wall-like structure size significantly; however, it does affect micropyramids shapes due to the plane selectivity and etching speed. The residue masking layer is strong and stable, which can withstand long time KOH etching. For a long time HF etching with less SiO_2_ left, the second layer mask can induce an inversed structures after long time etching in KOH solution. Making using of the residue as a second masking layer for controllable microstructure fabrication on Si substrate is a simple and useful method which will be used for fabrication of various structures applied in plasmonics, microfluidics and superhydrophobic surfaces in the future.
